# Pathology

**Published:** 1857-05

**Authors:** 


### PATHOLOGY.

INFLUENCE OF VARIATIONS OF ELECTRIC TENSION AS A CAUSE OF DISEASE. By *William Craig*, Surgeon, Ayr.—The monograph of Mr. Craig alluded to in the communication of Dr. Littell, as having been addressed to Dr. Hollingsworth, the late editor of the Examiner, was accompanied also by a letter from that gentleman, in which he expresses his surprise, "That men whose every-day pursuit is in the field of medical science, continually in contact with vital operations, and who are characterized as lovers of matter of fact, are content, in such an important matter as the remote cause of endemic and epidemic scourges which periodically devastate the human race, to believe in the agency of miasm, which miasm has never been identified, and appears to be a mere phantom of the brain. I am very glad," he continues, "to find that on your side of the Atlantic, other views begin to be entertained, and I am strongly under the conviction that the electric theory will yet be recognized as the true one, and that the researches in this direction will ultimately lead to the most satisfactory results. The American Continent is a much better field for making observations connected with endemic and epidemic diseases than the little island which constitutes Great Britain. Here we are confined to a small portion of the earth's surface, with a comparatively uniform temperature, and can see the operations of nature only in a very limited form. The small swamps in the fenny counties of England, exhibit the endemic phenomena in a very circumscribed degree, and on this account strike the mind of observers less forcibly than might be the case in situations where there is a large exposure of swamp under the influence of a nearly vertical sun."

His own paper, which was originally published in the London Medical Gazette for June, 1851, extends in double columns through some eighteen pages of that periodical, and its reprint entire would therefore occupy more space than we could conveniently spare; but inasmuch as it relates to a subject at once important, interesting, and novel, and is moreover strikingly confirmatory of the views put forth by Dr. Littell, we have thought that an abstract of it would not be unacceptable to the readers of the Review.

It is entitled, On the Influence of Variations of Electric Tension as a Cause of Disease; and sets out with the opinion that such variations on the various parts of the earth, act prejudicially on those animals which are placed on the portions of the earth thus affected. It is assumed as postulata, that heat and electricity are identical and convertible, that every atom of ponderable matter is surrounded by a little atmosphere of heat, and that it is through the agency of this element that attraction, and cohesion between the primary constituents of bodies are maintained. The gaseous bodies, whether in their aeriform state, as in the atmosphere, or solidified, as in vegetable combination, possess a great amount of latent heat, which is evolved in the new combination formed in the animal economy, and is the source of warmth to the system. The electricity so constantly and so liberally supplied by the various decomposing processes of respiration, digestion, and assimilation cannot, however, be intended merely for the support of animal temperature, but must have some other important work to perform, and what more likely than to minister to the vital operations in corporeal existence? The analogy, if not the identity of electricity and the nervous power is maintained, on the ground that the action of the one, can be successfully substituted for the other. Experiments showing, in the language of an able physiologist, "that a current of electricity sent along the referent nerves produces effects precisely analogous to those which are consequent on the transit of nervous forces. If it be sent along a motor nerve, muscular action is the result; along sensitive ones we effect the sensation peculiar to that nerve. Thus by means of a simple galvanic current passed through the eye, we produce the effect of light; through the auditory nerve, that of sound; and the nerves of smell and taste may be similarly acted upon." Dr. Wilson Philip has asserted that he can produce the secretion of the gastric juice by sending a current along the divided pneumogastrics.

This view is further confirmed by the structure and distribution of the nerves, as developed by the microscopical researches of MM. Prévost and Dumas, and the conviction is confidently expressed, not only that electricity evolved during respiration and assimilation is that which supplies nervous power, but that the structure of the nervous system favors the conclusion that the nervous forces are effected on the principle of a galvanic arrangement. Admitting the truth of this principle, it will follow that suspension or derangement of those provisions which nature has furnished for preserving a continual supply of vital electricity, cannot fail to affect the system prejudicially, in proportion to the amount of its abstraction.

The phenomena of disease prove that the first morbid impression is made upon the nervous system. The tumultuous form of nervous action which constitutes a rigor, conveys to those who are the subjects of it, the sensation as of a sudden abstraction of heat; coincident with which, there is a general derangement of the secretions, and a sudden failure of muscular power. Considering then that electricity and nervous force are identical, that the electricity evolved during the processes of respiration and assimilation is that which supplies the vital electricity to the nervous system, and that any cause which hinders the supply, or suddenly and to a great extent withdraws it after being supplied, must necessarily be mischievous, we have an intelligible combination of causes which will injuriously affect the system; without resorting to an imaginary miasm, which is not known as anything tangible, or appreciable by any of the senses, and which has eluded all search into its reality.

Taking cold will thus be an easily comprehensible idea. The escape of heat—that is the withdrawal of electricity from the body—is understood to be taking cold. The abstraction of vital electricity from a person whose nervous system has none to spare, will cause derangements that will be developed in some form of disease, the nervous currents in such circumstances, acting on a secreting gland, may be insufficient to elaborate from the blood those constituents which are required to form the various secretions; and in this manner the secretion may be imperfectly eliminated, the depuration of the blood incompletely effected; and the retention of those elements which ought to have been given off, will give rise to diseases which result from the vitiation of the fluids of the body.

Water in assuming the form of vapor absorbs a large quantity of electricity, and during this process portions of the earth, and the objects upon it are deprived of their due share. It is thus that injurious influences are exerted, especially on the predisposed, as are sufficient to cause epidemic and wide-spread disease.

In tropical countries the rain falls in greater quantity, and evaporation, which is effected by the radiation of solar heat, is consequently more active, carrying off the electrical fluid from the earth, and leaving it in a state of negative electricity. So constantly is humidity associated with the existence of endemic and epidemic diseases, that their extent and virulence, as a general rule, will be in proportion to the amount and rapidity of evaporation in any given situation. The rainy season, or the period immediately after it, when radiation and evaporation are greatest, is consequently the most sickly in tropical climates. The insalubrity of places in hot countries, where the sea-coast and rivulets are covered with mangrove vegetation, has been particularly observed, and is attributed to the peculiar nature of those bushes in growth and decay, absorbing moisture and facilitating evaporation. A constant drain of the electric fluid is thus kept up; and the electrical conditions of the animals being always positive, they suffer loss from the tendency of this fluid to maintain an equilibrium. In open and inland countries, destitute of marshes and jungle, the humidity is only occasional and of short continuance, and the insalubrity, therefore, is casual and temporary. The unhealthfulness of marshes is in proportion to the warmth of their position, and the consequent evaporation. On this principle it ought to follow that ague and other diseases which occur near marshes, should be mild or severe, just in proportion to the amount of evaporation. When the water scarcely covers the earth, the soil and plants become much more heated; and radiation and evaporation are consequently greater than when the ground is entirely overflown.

There might be cited from many writers on pestilential diseases in tropical climates, examples of wide-spread deadly disease, and at the same time an absence of every other apparent instrumentality. There was no vegetable or animal decomposition, or any other source of insalubrious effluvia, on mere sandy plains, but the speedy evaporation of the recently fallen rains, and the presence of a severe pestilential scourge.

Besides the effect of evaporation, there may be some occult influence in operation on the mineral strata that constitute the crust of the earth, of good-conducting power, which may disturb the regularity of the distribution, and unsettle the equilibrium of the electric fluid, withdrawing it probably into more central regions, and leaving the surface in a highly negative condition. In this way may be produced those occasional and epidemic attacks of pestilential disease, which cannot be attributed even to the existence of those circumstances which are generally looked upon as remote causes. That this is not mere hypothesis, is proved by the observations of M. Andrand, during the prevalence of cholera in Paris in 1849. They were made with a very powerful machine; and in a communication to the French Academy, dated on the 10th of July, of that year, he says: "I have remarked that since the invasion of cholera, I have not been able to produce, on any occasion, the same effect. Before its appearance, in ordinary weather, after two or three turns of the wheel, brilliant sparks of fire, of six centimetres in length, were given out. During the months of April and May, the sparks obtained, by great trouble, have never exceeded two or three centimetres, and their variations accorded very nearly with the variations of cholera.

"This was already for me a strong presumption that I was on the trace of the important fact I was endeavoring to find. Nevertheless, I was not quite convinced; because one might attribute the fact to the moisture that was in the air, or to the irregularities of the electric machine. Thus I waited with patience the arrival of fine weather, and heat, to continue my observations with more certainty. At last fine weather; and, to my astonishment, the machine, frequently consulted, far from showing, as it ought to have done, an augmentation of electricity, has given signs less and less sensible, to such a degree, that during the days of the 4th, 5th, and 6th of June, it was impossible to obtain anything but slight cracklings without sparks. On the 7th of June the machine remained quite dumb. This new decrease of the electric fluid has perfectly accorded with the renewed violence of the cholera, as is only too well known. For my own part, I was not more alarmed than astonished; my conviction was complete. At last, on the morning of the 8th, some feeble sparks reappeared, and from that hour the intensity decreased. Towards evening, a storm announced, at Paris, that the electricity had re-entered its domain; to my eyes, it was the cholera that disappeared with the cause which produced it. The next day I continued my observations; the machine, at the least touch, rendered with facility some lively sparks."

Experiments, with the same result, were carefully made in Glasgow, during the winter of 1849, when that city suffered from a similar visitation; and these facts, Mr. Craig regards as very conclusive in favor of the theory which he advocates. They distinctly indicate that the electric condition of the mineral strata and superincumbent mineral debris on which Paris and Glasgow rest, were, at the period when cholera raged, in a negative or low state of electric tension.

Besides these particular and occasional influences which operate on a large scale to produce epidemic and severe pestilential diseases, the occurrence of special and individual cases may be accounted for on the same principle. A person in impaired health, or declining years, is exposed to a shower of rain, and sits inactive until his clothes dry upon him. With just sufficient elimination of vital electricity to supply nervous currents, and none to spare in radiation to convert the water in his clothes to vapor, every particle of heat thus abstracted will be injurious. Similar exposure in a more vigorous state of health after severe exhaustion, would be followed by the same consequences, especially if the individual, in a state of perspiration, should imprudently sit or lie upon the ground—the greater conducting power of which would rapidly convey the electricity from the system.

The external covering provided for preserving the warmth of the inferior animals, gives further countenance to this theory. The hair, skin, and adipose tissue of quadrupeds, and the feathers which adorn and protect the fowls, are all good non-conductors of electricity. Man, less carefully guarded by nature, is endowed with faculties which teach him to protect himself; and non-conducting materials, as wool, hair, silk, &c., have always been selected as a defence from cold, apart from all philosophical considerations. The barbarous inhabitants of the torrid zone,—who can endure no other covering, besmear themselves with oil or grease for the same reason. During the prevalence of a very fatal fever at Bombay, it was observed that the natives employed in an oil establishment, whose bodies were always thus repulsively coated, continued in perfect health, though hundreds not so protected were dying all around them.

In tropical regions, more especially, vessels at sea are often attacked with severe and dangerous disease; and as this evidently cannot proceed from miasms or emanations from the soil, its origin must be sought for in some circumstances connected with the ship itself. The wood of which it is composed, is a good non-conductor, and the crew are thereby nearly insulated. But evaporation from frequent washings of the deck, or dampness from leakage or other cause, by which electricity is withdrawn from the system, furnishes a ready solution of the problem. Ships have suffered severely from yellow fever, while the decks were deluged with water several times a day, whereas others in which attention was given to keep everything dry, have been comparatively healthy.

The magnetic hills of the southern peninsula of India, especially those of Tavachymalle, are mentioned as a remarkable example of the irregularity and inequality of electric tension. There is nothing in their appearance to account for their unhealthfulness, and the cause can only be found in the character of the soil. This consists in a large proportion of ferruginous hornblende, which becomes highly magnetic; and it is supposed that the diminished amount of latent electricity in the disintegrated rocks, gives them a capacity of absorbing it from every object which comes in contact with them; and hence their insalubrity.

After some practical observations respecting clothing, the construction of houses, the selection of sites for building, &c., the bearing of the theory on the subject of contagion is next adverted to; and while the injurious effects of the concentrated emanations from the bodies of patients laboring under fever, are not denied, the belief is expressed that they are less influential than is commonly imagined, and that the diseases supposed to be produced by them, are more usually dependent on the altered electric relations of the body.

Vegetable life is maintained on the same principle as animal life, but in a less vigorous and more modified form; and the diseases which appear in this portion of organized structures, are produced in a somewhat similar manner. There is this difference, however, in the case of vegetable life, that free electricity is indispensable to its existence; whilst animal life would be supported quite independently of it.

We cannot follow Mr. Craig in all his reasonings and illustrations,—interesting and ingenious as they are. They strongly corroborate in many particulars the views set forth in the kindred essay of Dr. Littell; and the double witness thus borne by two separate observers, certainly strengthens its claim to the attentive consideration of the profession. We subjoin the recapitulation with which the pamphlet concludes.

"1st. That heat and electricity are identical, as the one can be converted into the other.

"2d. That a large volume of electricity surrounds every primary constituent of matter, especially that form of matter which constitutes the gaseous bodies.

"3d. That animal heat is supported by the electricity liberated from the primary constituents of matter during the processes of respiration, digestion, and assimilation.

"4th. That electricity is evolved during these processes on the same principle as that which is evolved during the action of a galvanic arrangement.

"5th. That electricity and nervous power are analogous, if not identical; as the action of the one can be successfully substituted for the other.

"6th. That the majority of diseases are caused either by the sudden abstraction, or slow subduction of electricity from the body.

"7th. That a low state of electric tension on the surface of the earth, produced either by the operation of evaporation, or some occult movement in the great internal currents of the earth, is the remote cause of epidemic and pestilential diseases.

"8th. That occasional and ordinary diseases are produced by the sudden abstraction or slow subduction of electricity from the body, or its undue elimination during the vital processes.

"9th. That since electricity is so essential to the integrity of the vital operations, it is indispensable that measures be taken to promote its evolution and prevent over-radiation.

"10th. That electricity is the source of vitality in vegetable life.

"11th. That electricity is attracted by the fibres of the roots of plants; and by the instrumentality of the electric fluid does the plant extract its constituents from the soil.

"12th. That vegetables of rapid growth require a large supply of electricity to secure their perfection and completion; and the potato is a plant of this kind.

"13th. That the disease in the potato was produced by want of nutrition.

"14th. That the want of nutrition arose from defective electric agency.

"15th. That the cause of the deficiency of this agency, was those abstracting agencies which produced low tension of electricity."

